# Quantitative Phenotyping of Duchenne Muscular Dystrophy Dogs by Comprehensive Gait Analysis and Overnight Activity Monitoring

**DOI:** 10.1371/journal.pone.0059875

**Published:** 2013-03-27

**Authors:** Jin-Hong Shin, Brian Greer, Chady H. Hakim, Zhongna Zhou, Yu-chia Chung, Ye Duan, Zhihai He, Dongsheng Duan

**Affiliations:** 1 Department of Molecular Microbiology and Immunology, School of Medicine, The University of Missouri, Columbia, Missouri, United States of America; 2 Department of Electrical and Computer Engineering, The University of Missouri, Columbia, Missouri, United States of America; 3 Department of Computer Science, The University of Missouri, Columbia, Missouri, United States of America; Medical College of Georgia, United States of America

## Abstract

The dystrophin-deficient dog is excellent large animal model for testing novel therapeutic modalities for Duchenne muscular dystrophy (DMD). Despite well-documented descriptions of dystrophic symptoms in these dogs, very few quantitative studies have been performed. Here, we developed a comprehensive set of non-invasive assays to quantify dog gait (stride length and speed), joint angle and limb mobility (for both forelimb and hind limb), and spontaneous activity at night. To validate these assays, we examined three 8-m-old mix-breed dystrophic dogs. We also included three age-matched siblings as the normal control. High-resolution video recorders were used to digitize dog walking and spontaneous movement at night. Stride speed and length were significantly decreased in affected dogs. The mobility of the limb segments (forearm, front foot, lower thigh, rear foot) and the carpus and hock joints was significantly reduced in dystrophic dogs. There was also a significant reduction of the movement in affected dogs during overnight monitoring. In summary, we have established a comprehensive set of outcome measures for clinical phenotyping of DMD dogs. These non-invasive end points would be valuable in monitoring disease progression and therapeutic efficacy in translational studies in the DMD dog model.

## Introduction

Duchenne muscular dystrophy (DMD) is an X-linked lethal muscle disease caused by mutations in the dystrophin gene [1]. While the majority of DMD studies have been performed in dystrophin-null mdx mice, the lack of severe clinical presentation in mdx mice has limited translation of findings from mice to human patients [2]. In contrast to mdx mice, dystrophin-deficient dogs were discovered because of their striking clinical symptoms such as an early onset of muscle weakness, muscle atrophy and stiff gait [3]–[5]. The majority of canine studies have used golden retriever muscular dystrophy (GRMD) dogs [3], [5]. In GRMD dogs, a point mutation in intron 6 results in erroneous bypass of exon 7 and subsequent premature translation termination [6]. Over the last twenty years, a number of different dystrophin-deficient dogs have been identified in other dog breeds (reviewed in [2], [7]). These new DMD canine models carry a variety of dystrophin gene mutations such as point mutation in intron 50 or exon 52, repetitive element insertion in intron 13 or 19, and whole gene deletion [2].

The inbreed mdx mice are highly homogenous in their genetic traits. It is very likely that the lack of genetic diversity in mdx mice has also contributed to the limited translational value of this model. In contrast to mdx mice, we have recently created dystrophic dogs that are on the mixed genetic background of golden retriever and Labrador retriever [8], [9]. These dogs may better reflect genetic heterogeneity in human DMD patients and more realistically illustrate human disease [2].

Several groups have recently begun to explore non-invasive methods (including accelerometry, kinematics and goniometry) to define clinical manifestations in the canine DMD model using GRMD dogs [10]–[13]. However, gait analysis has never been performed in other breeds of dystrophic dogs that carry a different mutation. Importantly, the published studies have only evaluated aspects of gait changes and a comprehensive analysis is lacking. It is well appreciated that confounding influences from the environment and investigators may introduce subjective bias in kinematic studies. Unfortunately, a truly objective method to evaluate spontaneous dog movement has not been developed. This study was designed to establish robust noninvasive clinical end points that can be used to quantify mobility changes in dystrophic dogs. Briefly, we recorded the gait pattern and voluntary activity of golden retriever/Labrador retriever hybrid dogs (three normal and three affected from the same litter) using high-resolution digital video recorders from three directions (side view, oblique view and top-down view). Information on the stride pattern (length, time and speed), joint angle and mobility of both forelimb and hind limb were obtained from the side and oblique view recording. Information on spontaneous whole body activity was obtained from overnight top-down view recording. We found that the stride length and stride speed were significantly decreased in affected dogs. The mobility of the limb segments and joints was also significantly reduced in affected dogs. Furthermore, affected dogs showed significantly less movement during overnight monitoring.

## Materials and Methods

### Animals

All animal experiments (including breeding, housing and rearing of the experimental subjects) were approved by the Animal Care and Use Committee of the University of Missouri and were performed in accordance with NIH guidelines. Experimental dogs were produced by artificial insemination crossing the semen from an affected Labrador muscular dystrophy (LMD) sire with a GRMD carrier dam [8], [14], [15]. LMD carries a repetitive element insertion in intron 19, which terminates dystrophin expression [8], [15]. All experimental dogs were from the same littler and evaluation was performed at the age of 8 months. The genotype was determined using established PCR protocols as we described before [8]. The diagnosis was further confirmed by the significantly elevated serum creatine kinase (CK) level in affected dogs. Information on experimental dogs is provided in Table 1. Dog height was measured using a metric ruler when a dog stood on a level surface on all four legs. The height between the withers and the ground level was defined as the dog height.

**Table 1:** Dog information.

Dog ID	Phenotype	Sex	Age (m)	Body weight(kg)	Dog height (cm)
D09-41	Normal	Male	8	30.3	58
D09-45	Normal	Male	8	28.3	49
D09-42	Normal	Male	8	35.7	56
D09-44	Normal	Male	8	29.7	46
D09-40	Affected	Male	8	16.7	53
D09-43	Affected	Female	8	18.1	51
D09-46	Affected	Female	8	17.1	NA

NA, not available.

### Video Recording Equipment and Software

High-definition XR-500V (Sony; Tokyo, Japan) digital video camcorders were used to record the movement of experimental subjects. This camcorder allows high-speed slow motion recoding at the speed of 120 frames/second. This camcorder is also equipped with the Sony G lens and the Exmor R™ CMOS sensor for videotaping at low light.

For gait study, recorded video clips were converted to digital images in the portable network graphic (PNG) format (120 image frames for every second video recording) using a free online software K-multimedia player (http://www.kmplayer.com/forums/). In each PNG image, the position of selected limb joints (see below) was marked manually using a customer macro on the NIH ImageJ software (version 1.45 h) (http://rsbweb.nih.gov/ij/index.html) by one investigator (BG). The marking was double checked by another investigator (JS). For overnight monitoring, recorded videos were converted into individual PNG image frame every 3 seconds. Difference between neighboring frames was analyzed with the NIH ImageJ software (version 1.45 h).

### Gait Study

**Figure 1:**
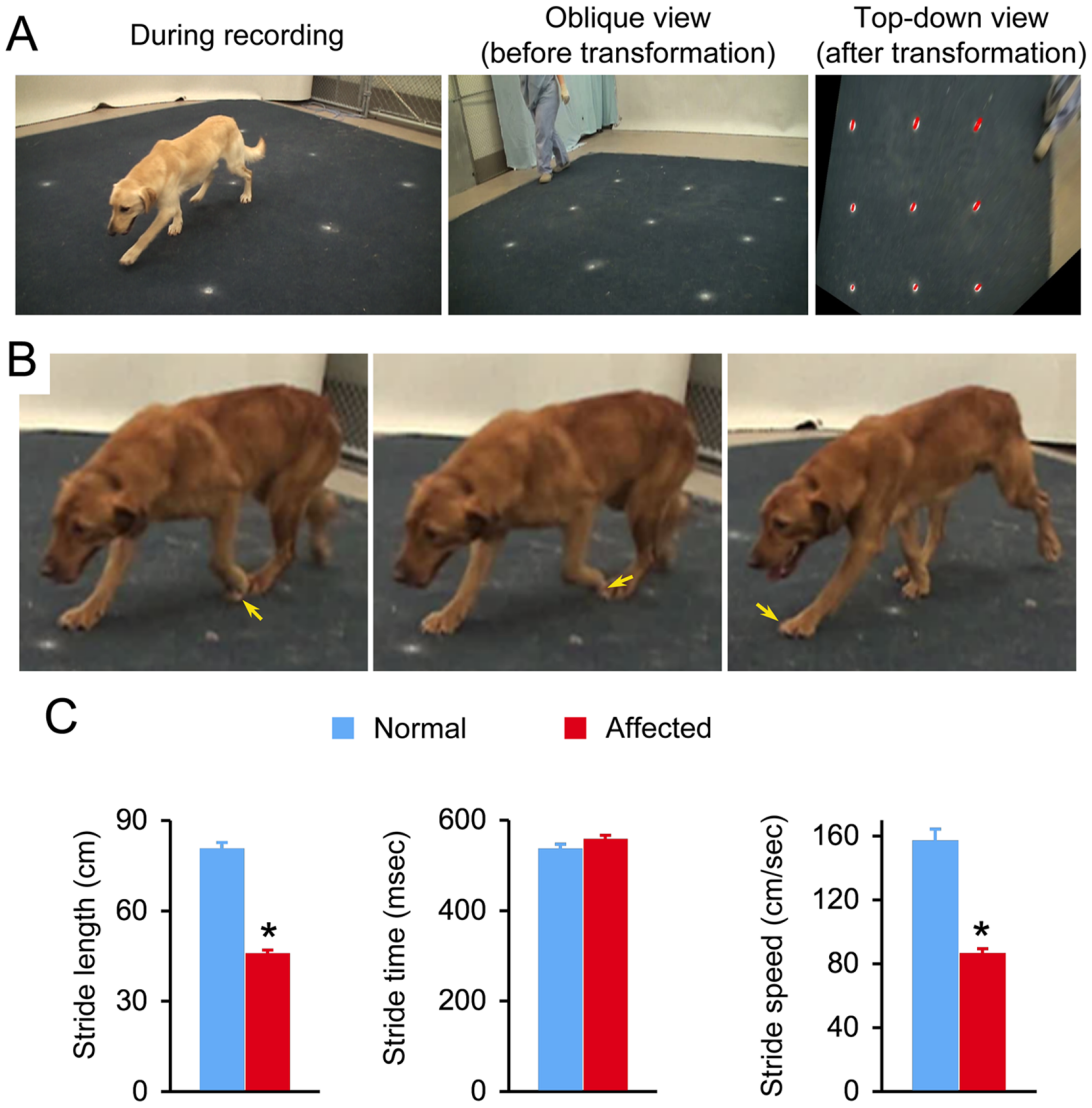
Quantitative evaluation of the stride properties. **A**, Overview of the video recording setup for stride measurement. Left panel, a dog during recording. Middle panel, oblique view of the mat with position markers. Right panel, transformed top-down view of the mat with position markers. **B,** Representative serial snap images of a dog obtained from oblique-view recordings. Arrow points to the position of the left forelimb paw from the time it lifts up from the ground (left panel), in the air (middle panel) and returns to the ground (right panel). **C,** The stride length and speed are significantly reduced in dystrophic dogs. Asterisk, significantly different between normal and affected dogs. Representative video clips from normal and affected dogs are shown in movies S1 and S2, respectively.

**Figure 2:**
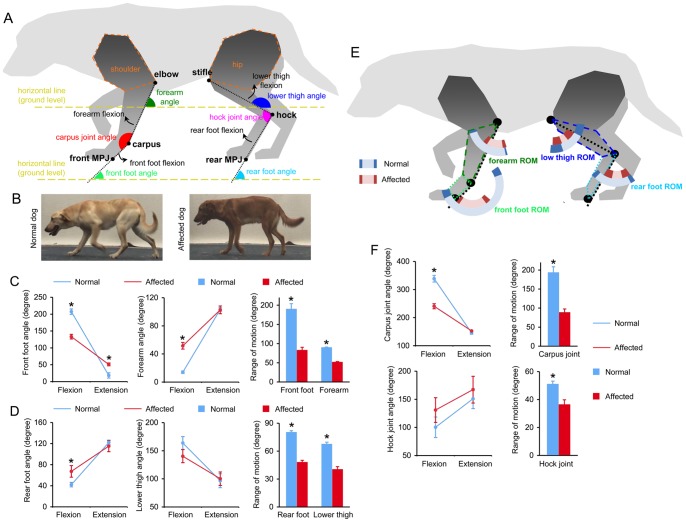
Angle measurements revealed significant reductions in the range of motion of the limb segments and joints in dystrophic dogs. **A**, Cartoon illustration of the reference points (elbow, carpus, front MPJ, stifle, hock and rear MPJ), limb angles (forearm angle, front foot angle, lower thigh angle and rear foot angle) and joint angles (carpus joint angle and hock joint angle). Also shown is the direction of limb segment flexion. The directions of carpus and hock joint flexions are the same as that of front foot and rear foot flexions, respectively. The shoulders and hips were not studied. **B,** Representative pictures converted from side-view video recording. Left panel, a normal dog; Right panel, an affected dog. The affected showed a stiff and straightened posture. **C,** The angles and the range of motions in the front foot and forearm. **D,** The angles and the range of motions in the rear foot and lower thigh. **E,** Doughnut drawing of the range of motion in each limb segment. Normal dogs, outer doughnut; Affected dogs, inner doughnut. Dark shaded region stands for the standard error of mean. The outline of each limb segment is color-coded to be consistent with the color of legend labels. **F,** The angles and the range of motion in the carpus and hock joints. Asterisk, significant difference between normal and affected dogs. Representative side-view video clips from normal and affected dogs are shown in movies S3 and S4, respectively.

Stride and joint angle were measured using two camcorders in a room equipped with a non-slippery mat. The mat was marked with fixed distance of 33 inches (This distance was arbitrarily chosen according to the size of the room) (Figure 1A). The markers were used as the reference points to convert the oblique view image to the top-down view image during data processing. The camcorder was placed on a tripod. Video recording was performed at 120 frames/sec. For stride measurement, video was taken from an oblique angle to ensure the best visualization of all four legs for the entire gait cycle (Figure 1B, Movies S1 and S2). For limb and joint angle measurement, a separate camcorder was used to record video from the dog’s side at the height of the stifle (Figure 2A and B, Movies S3 and S4).

For gait study, all subjects were trained for unleashed walking and accustomed to the test environment for one week. On the day of test, dogs were allowed to walk freely on call. Only the video clips that showed dog walking (more than two limbs are always touching ground and each limb moves one after another) were used for gait analysis. Recordings of obvious trotting (two limbs at diagonal ends of the body move as a set, tossing the weight to each other) or galloping (two forelimbs and two hind limbs move in pair respectively and all limbs are suspended for a significant amount of time after the last-in-sequence limb leaves the ground) were excluded in the analysis. For stride measurement, the oblique view recording was first transformed into top-down view images based on the positions marked on the mat (Figure 1A). A stride was defined as the frames starting from the time the toe tip left the ground to the time the same toe tip touched the ground again (Figure 1B).

Limb and joint angle measurements were obtained only from side-view video clips from the side facing the camcorder. Video clips in which the dog did not walk strictly along the center of the mat were not used in the analysis. To accurately determine angle changes, we intentionally excluded the shoulder and hip in the analysis because their anatomic points cannot be clearly discerned on images converted from video clips (Figure 2A). The following three reference points were used for the forelimb (1) elbow, the caudal point that makes the angle at the elbow; (2) carpus, the caudal point that makes the angle at the carpus; and (3) front metacarpo-phalangeal joint (MPJ), the most caudal point touching the ground or the angled point when the limb is in the air (Figure 2A). The following three reference points were used for the hind limb (1) stifle, the cranial point that makes the angle at the stifle; (2) hock, the caudal point that makes the angle at the hock; and (3) rear MPJ, the most caudal point touching the ground or the angled point when the limb is in the air (Figure 2A).

To determine the range of the motion (ROM) of the forearm (the line between the elbow and the carpus), front foot (the line between the carpus and the front MPJ), lower thigh (the line between the stifle and the hock) and rear foot (the line between the hock and the rear MPJ), we arbitrarily defined their angles using the ground (horizontal line) as the reference level (Figure 2A). Specifically, the forearm angle is defined as the angle formed between the elbow-carpus line and the ground level line on the caudal side. The front foot angle is defined as the angle formed between the carpus-front MPJ line and the ground level line on the caudal side. The lower thigh angle is defined as the angle formed between the stifle-hock line and the ground level line on the caudal side. The rear foot angle is defined as the angle formed between the hock-rear MPJ line and the ground level line on the caudal side. The ROM for each limb segment was then calculated using the formula [ROM] = [the largest value obtained from angle measurement] - [the smallest value obtained from angle measurement].

Based on limb angle measurement, we also calculated angles for the carpus and hock joints. Specifically, the carpus joint angle (the angle formed between the elbow-carpus line and the carpus-front MPJ line on the cranial side) was calculated using the formula [carpus joint angle] = 180°−[forearm angle]+[front foot angle]. The hock joint angle (the angle formed between the stifle-hock line and the hock-rear MPJ line on the cranial side) was calculated using the formula [hock joint angle] = 180°−[lower thigh angle]+[rear foot angle]. The ROM for each joint was then calculated using the formula [ROM] = [the largest value of the calculated joint angle] - [the smallest value of the calculated joint angle].

To minimize variation from single recording session, each experimental subject was recorded three times on three different days. Data from all three recordings were used for analysis.

### Overnight Monitoring

**Figure 3:**
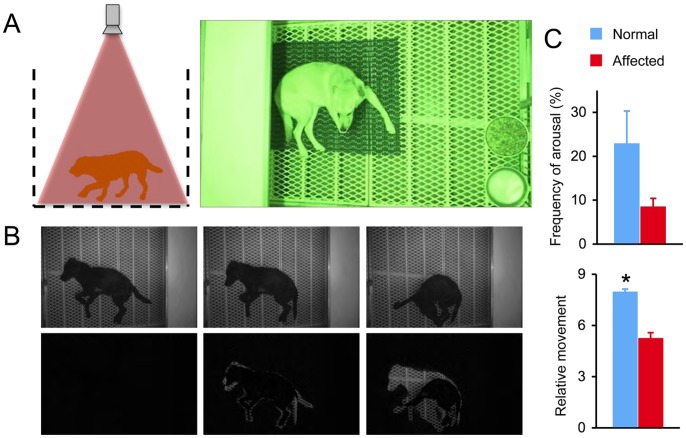
Dystrophic dogs show reduced activity during overnight monitoring. **A**, The set up of the overnight video monitoring system. Left panel, a cartoon illustration showing the position of the dog and the camcorder. The video recording was taken from the top of the cage to allow full visualization of the entire floor of the cage. Right panel, a representative image of a dog during overnight monitoring. **B,** Representative snap images at different time points of recording in a dog. Top panels, the original recording; Bottom panels, after image processing. Left panel, when the dog did not change position, the processed image shows no signal (only dark background). Middle and right panels, when the dog changed the position, the difference between the current and the previous (3 sec prior; see Method for detail) images is revealed as white/gray signals within the dark background. **C,** Quantification of the frequency of arousal and the relative movement. Representative overnight monitoring video clips from normal and affected dogs are shown in movie S5.

Each dog was housed in its individual run and evaluated separately. The camcorder was placed on a custom-made mounting bracket that sits on the top of the cage to ensure top-down view of the whole cage (Figure 3A). The size of the dog cage used in this study was 46 inch×77 inch. However, the protocol is applicable to a cage of any size. Overnight activity of the dog was monitored between 7 pm and 5 am under low-lux light. During this period, there was minimal interference from environment cues and animal caregivers. Movement was defined when the displacement exceeded the background variation (Figure 3B). Frequency of the movement and the amount of movement were deduced from the processed images (Movie S5). Similar to the gait study, overnight monitoring data for each dog were also from three independent recordings at three different nights.

### Statistical Analysis

Data are presented as mean ± standard error of mean. Statistical difference between normal and affected dogs was assessed with student t test. A p<0.05 was considered statistically significant.

## Results

Experimental dogs were generated by crossing an affected Labrador retriever male with a golden retriever carrier female. Offspring were thus on a mixed genetic background. At the time of the study, dystrophic symptoms (such as muscle atrophy and joint contracture) were readily recognizable in all affected dogs. Affected dogs also showed less activity when released from the cage.

The affected dogs showed a significantly reduced stride length (affected, 45.4±1.5 cm; normal, 80.2±2.5 cm; p<0.00001) (Figure 1C). Stride speed was also significantly reduced in affected dogs (affected, 85.9±3.6 cm/sec; normal, 156.5±7.9 cm/sec; p<0. 0.00001) (Figure 1C). Interestingly, stride time was not altered in dystrophic dogs (Figure 1C).

Limb angles were determined from side-view recording (Figures 2). The forearm and rear foot of affected dogs showed a flexion angle significantly larger than that of normal dogs, suggesting that these limb segments of affected dogs cannot swing forward as much as that of normal dogs (forearm flexion angle: affected, 51.7±4.7°, normal 13.9±1.8°; rear foot flexion angle: affected, 67.3±11.0° and normal 42.1±4.4°; p<0.01) (Figure 2C and 2D). The front foot showed the most dramatic changes. The front foot of affected dogs had a significantly smaller flexion angle (affected, 133.2±6.8°; normal, 207.3±8.1°) and a significantly increased extension angle (affected, 51.0±4.3°; normal, 18.3±7.9°). These changes suggest that the mobility of the front foot was markedly restricted in affected dogs compared to that of normal dogs (Figure 2C). Interestingly, among all limb angles studied, significant differences were more frequently seen in the action of flexion (angles of forearm, front foot and rear foot) than extension (only the front foot angle). There was no difference between normal and affected dogs in the lower thigh angle. Importantly, all studied limb segments of dystrophic dogs showed a significant reduction in their ROM (Figure 2C, D, E).

The carpus and hock joint angles were deduced from the limb angle. Only the flexion angle of the carpus joint showed a significant difference between normal and affected dogs. Nevertheless, the ROM was significantly decreased in both joints in affected dogs (Figure 2F).

There was relatively little movement during overnight activity monitoring. Normal dogs changed position in approximately 20% of the observation time, while affected dogs only changed position in less than 10% of the observation time. Although the frequency of arousal was less often in affected dogs, it did not reach statistical significance (Figure 3C). The amount of movement during the night was significantly reduced (by approximately 40%) in affected dogs, as compared to normal dogs (Figure 3C, Movie S5).

## Discussion

Abnormal gait is a major clinical manifestation in DMD patients [16]–[18]. Similar symptoms are also frequently seen in dystrophin-deficient dogs. Unfortunately, quantitative evaluation of gait changes in dystrophic dogs has not been performed until recently. Marsh et al recorded side-view video of walking dystrophic dogs and described hind limb movement using a kinematic method [10]. Barthelemy et al studied dog movement using an accelerometer [11], [12]. Gaiad et al determined the range of the motion of the joints in dystrophic dogs by manually measuring the jointing angle with a goniometer [13]. While these studies have provided useful information, there are also important limitations. For example, in the Marsh et al all study, all of the data were collected in one day with minimal acclimation of the subjects to the experimental environment. Some techniques used by the authors (such as large diffuse reference points and the low-speed camcorder) may have also introduced inaccuracy [10]. In the Barthelemy et al study, the authors used accelerometry. However, this method cannot provide information on the joint angle and limb ROM [11], [12]. In the Gaiad et al study, the data on the joint angle was obtained from stationary dogs rather than during dog movement [13]. It is also worth pointing out that none of the published studies were performed on dogs of the same age, neither have these studies considered potential interferences from investigators and the experimental environment. Finally, all published studies were performed in GRMD dogs that were all originated from a single affected male.

We recently obtained a litter of four wild type and three affected dogs from a single breeding [8]. Since this was a cross between a LMD male and a GRMD carrier, all offspring should be on the same hybrid background of 50% Labrador and 50% golden retriever. These siblings provided a valuable opportunity to compare mix-breed normal and affected dogs in an age- and genetic background-matched manner. To this end, we conducted a non-invasive video quantification study in three wild type and three affected dogs (the fourth wild type dog of the litter was not available for study). To gain a comprehensive evaluation, we used three different video recording perspectives, including oblique view, side view and a top-down view used during overnight recording. Oblique view recordings, while dogs were walking, were transformed to top-down view images to permit accurate measurement of the stride properties (length, time and speed) (Figure 1A).

Analysis of oblique view recording revealed an approximately 40–50% reduction in the stride length and speed in affected dogs (Figure 1C). Our results are in line with those reported by Marsh et al and Barthelemy et al [10], [11]. Barthelemy et al found that both stride length and stride speed were reduced by approximately 50% in 2 to 9-month-old affected dogs [11]. Marsh et al compared male affected dogs with female carrier dogs (age range: 17- to 40-month-old) and they also noticed an approximately 40% reduction of the stride speed in affected dogs [10]. Taken together, consistent results from three different laboratories suggest that the stride length and stride speed represent reliable biomarkers to monitor muscle disease in dystrophic dogs.

Side view offers the best visualization of limb and joint angles (Figure 2). Since the positions of the forearm, front foot, lower thigh and rear foot were readily recognizable in digitized pictures obtained from video recording, we quantified angles formed by these limb segments and the ground (horizontal line) (Figure 2A). Based on these measurements, we also deduced angles of the carpus joint and hock joint as well as the ROM for four limb segments and two joints (Figure 2C–F). Marsh et al previously reported that the ROM was significantly reduced in the ankle (hock) joint in dystrophic dogs [10]. However, the ROM in the carpus joint was not studied, neither was the ROM in any limb segments studied. Here, we found that all of the ROMs of all the limb segments and joints that we examined were significantly reduced in dystrophic dogs. Hence, our results have substantially extended the observation of Marsh et al and provided more quantitative evidence that mobility is severely impaired in limbs and joints in affected dogs. Despite consistent findings in the ROM, measurements of the limb segment angle and joint angle revealed some surprising findings. Specifically, of all the angles studied significant differences between normal and affected dogs occurred mainly during flexion (Figure 2C, D, and F). Further, no differences were found in the angles of the lower thigh and the hock joint. We currently have no explanation for these intriguing results. We suspect that it may be attributed to differences in disease progression in different limb muscles.

One of the most important achievements of our study is the development of an overnight dog activity monitoring system. A major drawback of previously described methods (such as kinematics and accelerometry) is the potential confounding influences of the experimental environment and the investigators [10]–[12]. The overnight video recording assay described here overcomes these drawbacks and allows a true objective evaluation of spontaneous motion activity of a dog, albeit during a relatively rest period (Figure 3). As expected, affected dogs were indeed less active at night. They showed significantly less movement than normal controls (Figure 3).

It should be point out that in order to match with the age and genetic background, we were not able to achieve a perfect gender match between normal and dystrophic dogs. Specifically, two of the affected dogs used in the study were female dogs. Although we cannot completely exclude a potential influence of the gender on dog movement, we believe that such influence is likely minimal in the context of our study because there is no dramatic difference in the body weight and height between the male and female affected dogs (Table 1).

Collectively, our results suggest that there are quantifiable differences in the gait pattern and activity between normal and affected dogs. These non-invasive end points would be valuable in studies that assess disease progression and critical therapeutic interventions designed to improve muscle function in the canine model of DMD.

## Supporting Information

Movie S1 **A representative oblique view video clip of a normal dog showing a normal stride pattern.**
(AVI)

Movie S2 **A representative oblique view video clip of an affected dog showing a dystrophic stride pattern.**
(AVI)

Movie S3 **A representative side view video clip showing limb and joint angle in a normal dog.**
(AVI)

Movie S4 **A representative side view video clip showing reduced limb and joint mobility of an affected dog.**
(AVI)

Movie S5 **Representative overnight video clips from a normal (left-side) and an affected (right-side) dog during 3 hours of monitoring (For viewing purpose, the video recording was compressed).** Top panels, the original recording; Bottom panels, after image processing.
(AVI)
